# Empowerment or Exposure? Digital Literacy and the Vulnerabilities of Trust in Strangers Among Older Adults: Evidence from China

**DOI:** 10.3390/bs16040497

**Published:** 2026-03-27

**Authors:** Kaixuan Gao, Hui Zhang, Zeming Cheng, Bin Tang

**Affiliations:** 1 The Library, Shaanxi Normal University, Xi’an 710119, China; kaixuangao@snnu.edu.cn; 2 Office of Human Resources, Peking University, Beijing 100871, China; zhanghui@pku.edu.cn; 3 Center for Experimental Economics in Education, Faculty of Education, Shaanxi Normal University, Xi’an 710119, China; chengzeming@snnu.edu.cn

**Keywords:** digital literacy, trust vulnerability, older adults, digital risk society, ageing and technology

## Abstract

Digital literacy is widely promoted as enabling later-life inclusion, but its potential to generate trust-related vulnerabilities remains insufficiently examined, particularly in rapidly ageing societies. Using nationally representative data from the 2022 China Family Panel Studies including 1583 adults aged 60 and above, this study examines whether digital literacy is associated with older adults’ trust in strangers, which is interpreted here as a form of trust vulnerability in low-verifiability interactions. In this study, trust vulnerability is operationalised through respondents’ self-reported trust in strangers, while digital literacy is operationalised through behavioural competences spanning information and data literacy, communication and collaboration, content creation, digital transactions, and problem-solving. OLS regression models with Huber–White robust standard errors are employed. The findings indicate that higher levels of digital literacy are positively associated with stronger trust in strangers, suggesting that competence may heighten older adults’ inclination to trust and increase exposure to digital risks. This association is driven primarily by functional competences, with mobile payment use and problem-solving skills showing the largest and most consistent associations. By contrast, subjective well-being and perceived platform security show no significant mediating roles. The study recommends integrating functional training, trust calibration and risk-recognition education, alongside interpretable and verifiable safeguards that make security more visible to older adults.

## 1. Introduction

Population ageing and digital transformation are two of the defining forces of the twenty-first century. Technology can help older adults to access important resources and limit feelings of social isolation. However, increased access to technology means older adults are exposed to more privacy breaches, identity theft, and online scams (Lazarus et al., 2025). China sits at the intersection of these two global transformations. Official statistics indicate that by the end of 2024, the population aged 60 and above in China had reached 310.31 million (22.0%), including 220.23 million aged 65 and above (15.6%) (National Bureau of Statistics of China, 2025). At the same time, platform-based services and mobile digital infrastructures have become deeply embedded in everyday life. As of June 2025, China had approximately 1.123 billion internet users, of whom 1.022 billion used online payment services, representing 91.0% of all internet users (China Internet Network Information Center [CNNIC], 2025). Taken together, these trends substantially increase the salience of digital systems for older adults’ everyday participation, while also shaping the conditions under which digital engagement may generate both empowerment and new forms of vulnerability.

Older adults are at increased risk of attacks online as a result of generally lower levels of digital literacy, as well as reduced perception of risk and greater willingness to trust someone online (Havers et al., 2024). In digital environments, trust among older adults exhibits a marked paradox. In everyday social life, generalised trust is commonly regarded as a form of positive social capital that facilitates cooperation and social functioning. By contrast, in loosely regulated digital contexts, trust in strangers may be more easily exploited, thereby increasing older adults’ exposure to fraud, misinformation, and other forms of harm (Dai et al., 2022). Digital inclusion has become a central objective on the global policy agenda, with growing recognition that empowerment through digital participation must be accompanied by adequate protection, particularly for high-risk groups such as older adults (United Nations Development Programme, 2024). As a result, the relationship between trust and digital literacy has emerged as a key scholarly concern at the intersection of population ageing and digital transformation. A core question remains contested: for older adults, does the enhancement of digital literacy primarily operate as a mechanism of social empowerment, or does it, under certain conditions, coincide with new forms of risk exposure and trust vulnerability?

Across national and international frameworks, there is broad convergence around the core idea of digital literacy. It is understood not merely as the ability to perform technical operations, but as the capacity to use digital technologies in safe, critical, and responsible ways, particularly when individuals face uncertainty and potential risks (Organisation for Economic Co-Operation and Development, 2023). This understanding is reflected in the EU’s DigComp 3.0 framework, the OECD digital literacy agenda, and related United Nations policy documents. Drawing on this cross-framework consensus, our study adopts DigComp 3.0 as a domain-level framework to organise key dimensions of digital competence.

The role of digital literacy is depicted differently in existing studies. Some earlier works linked higher levels of digital competence to improved well-being, including social connectedness and more generalised trust, and also suggested that it may reduce susceptibility to fraud by strengthening confidence, perceived control, and resilience (Hofer et al., 2019; Zheng & Zhang, 2023; Choung et al., 2023; Jumrana & Ibrahim, 2023). However, subsequent research has brought the risks of enhanced digital participation into focus, as it increases the level of (mis)information encountered, phishing, and financial scams (Pehlivanoglu et al., 2024). Others fear that intensive online contact could trigger trust fragility, in which older adults may be overly trusting of what looks to them like a friendly user interface or persuasive design cue (Liu et al., 2021; Rossi et al., 2024). Recent discussions have stepped back from this binary analysis, arguing that digital literacy effects are context-specific and that benefits become more evident in nurturing systems, while hazards and risks are observed when digital environments are unregulated (Tinmaz et al., 2022). However, the existing evidence remains fragmented and still insufficient to offer a coherent account of how digital literacy relates to users’ trust susceptibility across different contexts.

These mixed findings raise a deeper conceptual question about trust in digital risk settings. Theoretical debates in risk society research present trust as fragile, suggesting that trust may entail forms of vulnerability under conditions of uncertainty (Beck, 1992; Szerszynski, 1999). But those debates have been little incorporated into empirical research in this regard. Many studies still conceptualise generalised trust as social capital, ignoring the possibility that generalised trust in strangers may be counterproductive in the digital risk society, such as in the presence of scams and misinformation. With regard to explanatory mechanisms, prior empirical studies have tended to focus predominantly on psychological pathways, most commonly subjective well-being and perceived safety, when examining the consequences of digital literacy (Xin et al., 2025). However, functional competences, such as information acquisition, problem-solving, and transactional skills, may directly shape privacy protection and risk perception (Büchi et al., 2017). This study seeks to integrate these perspectives within a unified analytical framework. At the same time, empirical coverage remains limited. Much of the available evidence is concentrated in high-income Western countries and often relies on small or local samples, leaving rapidly ageing societies such as China underexplored (Palmisano & Sacchi, 2024).

In this study, we draw on nationally representative data from the 2022 China Family Panel Studies (CFPS 2022) to examine how digital literacy is associated with trust vulnerability among older adults in China. Trust in strangers is interpreted here as a situational vulnerability that may arise in digitally mediated interactions, where verification cues are often limited and interactions frequently occur with unfamiliar others. With this framework, we further consider whether this association may operate indirectly through subjective well-being and perceived platform security. Accordingly, we expect digital literacy to be positively associated with trust vulnerability (H1), with subjective well-being (H2) and perceived platform security (H3) serving as potential mediating pathways. We also examine whether these relationships vary across competence domains and subgroups. Our study makes two distinct contributions. First, empirically, it draws on large-scale, nationally representative CFPS 2022 data with novel measures of digital competences, generalised trust, well-being, and perceived platform security, providing evidence from China on how digital literacy relates to trust vulnerability in a developing-country context and extending debates on ageing and digital inclusion across different institutional contexts. Second, it advances existing discussions by highlighting the role of functional competences and conceptualising digital literacy as both empowering and risk-generating, thereby broadening prior research that has focused on psychological mechanisms such as well-being and perceived safety.

## 2. Literature Review

Digital literacy has become a fundamental competence of active ageing, enabling older adults to access services, maintain social connections, and participate in the digital society. With the accelerated evolution of digital infrastructures, new forms of risk, such as privacy leakage, misinformation, and financial fraud, are becoming more and more prominent. In such an environment, how digital literacy is associated with individual risk perception and trust judgement in uncertain situations has become the focus of attention in the academic and policy fields (Beck, 1992; Sundberg, 2024). Understanding the tension between “empowerment” and “exposure” of digital literacy is of great theoretical and practical significance for assessing its relationship with the digital life of the elderly.

To provide theoretical background for this study, the review is organised into three strands: (1) research on digital literacy and older adults’ well-being; (2) research on digital literacy and trust in digital environments; and (3) studies that elaborate psychological, functional, and institutional pathways linking digital literacy to trust-related vulnerability.

### 2.1. Digital Literacy and Well-Being: From Empowerment to Psychosocial Gains

Most studies show a positive link between digital literacy and subjective well-being, with higher digital skills associated with greater life satisfaction, reduced loneliness, and better social engagement (Lee-Geiller, 2024). Such evidence suggests that older adults can turn digital access into more tangible outcomes for quality of life (Carrasco-Dajer et al., 2024). Other research also shows that the more digitally skilled individuals are more likely to engage in positive online behaviour, providing further evidence for digital inclusion and broader social integration (Livingstone et al., 2023). In China, internet use is closely tied to greater happiness, with more digitally active older adults reporting higher life satisfaction (Cheng et al., 2024).

Subjective well-being is theoretically defensible as a mediating pathway linking digital literacy to social trust among older adults. Existing studies show that digital literacy and internet use can enhance older adults’ well-being by improving social connectedness, reducing loneliness, strengthening intergenerational ties, and increasing perceived competence in everyday life (Soundararajan et al., 2023; J. Li & Zhou, 2021; Yan et al., 2023). In turn, higher subjective well-being has been associated with more positive evaluations of the social world and higher levels of social trust, with longitudinal evidence suggesting that subjective well-being may even precede subsequent increases in trust (Glatz & Schwerdtfeger, 2022; R. J. Zhang, 2020). Therefore, subjective well-being can be understood as a plausible psychological mechanism through which digital literacy reshapes older adults’ trust orientations, even if the magnitude of this pathway may vary across institutional and digital contexts.

Even so, the process of digital empowerment incurs expenses. For the elderly, improper access or use of information is frequently accompanied by higher risks, making them more vulnerable to problems such as fraud, infringement and privacy leakage (Y. Li et al., 2024). This suggests that elderly people may sometimes develop an illusory sense of security in online settings that in fact require heightened vigilance (Moran, 2020). There is a paradoxical relationship between digital empowerment and risk vulnerability, which shows that the understanding of digital capabilities should not be limited to skill analysis at the individual level, but should deeply reveal its inherent tension and risk mechanism in the institutional structure and cultural context.

This underscores the importance of supporting infrastructures and protective measures. Digital inclusion benefits depend on whether accompanying safeguards, service design, and governance systems evolve alongside increased participation. Without these supports, greater digital competence can coexist with heightened vulnerability, where psychosocial gains may not lead to reduced exposure to risks.

### 2.2. Digital Literacy and Trust: The Paradox of Empowerment in Digital Risk Societies

The existing research on the relationship between digital literacy and trust is mainly discussed from the three analytical dimensions of system, platform and society. From an institutional perspective, scholars have found that individuals with high digital operation ability can more effectively understand government services and e-government platforms, thus reducing institutional ambiguity and operational obstacles, and enhancing trust in public institutions (Lee-Geiller, 2024). On the platform level, especially in high-frequency use situations such as e-commerce and mobile payment, the accumulation of digital knowledge enables users to better understand the operation mechanism of the system, improve controllability and predictability, thereby reducing uncertainty and enhancing the willingness to use (Singh et al., 2024). At the social level, digital interaction promotes the flow of information between individuals and the accumulation of social capital in the virtual social network, thus improving the level of universal trust (Zhou et al., 2022). These studies suggest that digital literacy is often regarded as an “empowerment ability” associated with individuals’ sense of trust.

Theoretically, trust itself is inherently ambivalent. Classical trust theory emphasises that trust inherently involves the acceptance of uncertainty and vulnerability. Trust has been widely defined as the willingness to be vulnerable to the actions of another based on the expectation that the other will perform a particular action important to the trustor (Mayer et al., 1995), and has also been conceptualised as a mechanism that enables individuals to act under conditions of incomplete information by accepting potential risk (Luhmann, 2018). Extending this perspective to interactions among unfamiliar others, research has noted that trusting strangers involves the possibility of exploitation when reliable verification cues are absent (Thomson et al., 2015). In digitally mediated environments, interactions often occur with unfamiliar others and limited contextual cues, which can make verification more difficult and thereby render the vulnerability inherent in trust more salient (Lucassen & Schraagen, 2013; Thomson et al., 2015).

The risk society theory further highlights this duality. Beck pointed out that modern society is increasingly dependent on trust in managing complex technology systems, but when the protection mechanism fails, such dependence may also create new forms of risk (Beck, 1992). In the contemporary digital society, trust may become entangled with identity theft, online fraud and false information. These risks may be particularly pronounced among older adults, who often encounter greater difficulties when attempting to understand and apply digital protection mechanisms (Chen, 2025; Ebner et al., 2023).

Despite theoretical discussions on this paradox, empirical research remains limited, particularly in non-Western contexts undergoing rapid digitalisation. Existing studies often emphasise the empowering role of digital competence, while paying less attention to how digital participation may simultaneously reshape trust-related vulnerabilities. The question remains: does improved digital literacy reduce uncertainty and enhance resilience, or does it increase exposure to trust-related risks? This issue is crucial for shaping digital inclusion and security policies for the elderly, and this paper aims to address it systematically.

### 2.3. The Mechanisms Linking Digital Literacy to Trust Vulnerability: Psychological, Functional and Institutional Pathways

While there has been little focus on detailed mechanisms for explaining the relationship between digital literacy and trust among older adults, there have been strands of work drawing together empirically observed relationships. One line of research emphasises psychological mediation. Studies empirically demonstrate how digital competences can lead to greater subjective well-being and perceived control, which in turn predict reduced perceptions of risk. Empirical evidence from low-income older adult intervention programmes in Singapore suggests that the social use of digital technology is positively associated with well-being and quality of life (Hofer et al., 2019; Soundararajan et al., 2023). These findings suggest that digital literacy may influence trust vulnerability partly through psychological resources rather than through access alone.

In addition to psychological explanations, there has been recent evidence that functional competences (like accessing information, solving problems, and using ICTs for transactions) may also affect how people protect themselves and how they think about risk, independently of psychological mediators. In a nationwide survey on online communications, digital literacy outperforms attitudes in a wide range of online privacy-protective behaviours and thus directly affects private decision-making, independent of attitudes (Park, 2011). Recent survey evidence targeting older populations also shows that higher digital skills are linked to lower perceived risk and to heavier ICT usage in everyday life (Aleti et al., 2025), providing evidence for a functional modulating effect of skills on the distribution of trust and oversight. However, the prior work is still fragmented, and functional pathways are analysed in standalone fields such as privacy and risk-taking in daily life, as opposed to within a unified explanatory narrative.

A third perspective focuses on institutional mechanisms. Research into E-government reveals that when people see official digital mechanisms as useful and effective, they have more trust in their public institutions (Porumbescu, 2016). Recent research reveals that digital literacy is a moderating factor: more competent people benefit more from their perceptions of public institutions working digitally (Lee-Geiller, 2024). The literature on digital ecosystems also points to the fact that perceived security, accountability mechanisms, and intermediary governance play an important role in shaping user trust and dependence on digital platforms (Warner-Søderholm et al., 2018). Even though this body of literature acknowledges institutions as one of the critical mechanisms involved in trust, there is a lack of explicit focus on older adults in such contexts.

Despite these insights, the relative importance of these pathways remains unclear. Existing studies often emphasise the empowering role of digital competence while paying less attention to how digital participation may simultaneously reshape trust-related vulnerability. This limitation is particularly evident in rapidly ageing, non-Western contexts, where digital participation is expanding under distinctive institutional and cultural conditions.

### 2.4. Theoretical Framework

Based on the research gaps found in the above part, the theoretical framework of our research is based on two types of complementary literature: the theory of trust and risk and the theory of digital inclusion. The theory of trust and risk defines trust as a relational commitment established in uncertainty and potential risks, requiring individuals to operate in the absence of complete information and verification (Beck, 1992; Luhmann, 2018). In the digital media environment, this uncertainty is exacerbated by information asymmetry, anonymity and limited accountability, making trust a basic resource for action and possibly a source of vulnerability, especially for the elderly.

Digital inclusion theory, in turn, frames access to and use of digital technologies as shaped by underlying social inequalities and institutional conditions. While digital competences can enhance participation, autonomy, and agency, they may also reproduce or reconfigure vulnerabilities when protective infrastructures, regulatory safeguards, and risk literacy do not keep pace with participation expansion (Pérez-Escolar & Canet, 2023).

Building on these perspectives, this study integrates trust and inclusion theories to develop an analytical model that explains how digital literacy may simultaneously generate empowerment and trust-related vulnerability among older adults.

**Figure 1:**
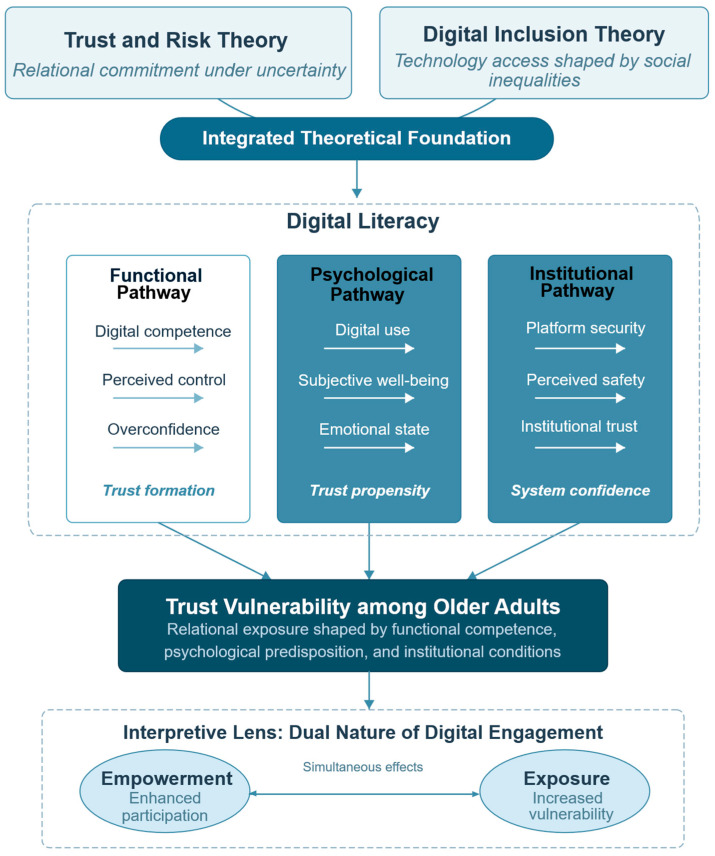
Theoretical framework linking digital literacy to trust vulnerability among older adults. Note: The framework integrates trust and risk theory with digital inclusion theory to illustrate how digital literacy is related to trust vulnerability through functional, psychological and institutional pathways. It highlights the dual implications of digital inclusion: empowerment and exposure.

Figure 1 illustrates the theoretical model developed in this study. Digital literacy is the centre point in Figure 1 that connects digital competencies with relational modes of vulnerability. We found three sets of related mechanisms through which digital literacy mediates trust. The functional route emphasises competence with behaviours and perceiving having control: competencies related to finding information, reasoning and transactions online may increase effectiveness and independence (Aleti et al., 2025; Lee et al., 2022), yet conversely also lead to excessive self-satisfaction when interacting with newly encountered strangers. The psychological path indicates that the effects of online skills are through subjective well-being, because online skills are correlated with higher life satisfaction, self-confidence, and perceived control, which predict the probability to trust online (Ji et al., 2024; S. Li, 2025). The institutional route positions trust within institutional arrangements through the perceptions of platform security, privacy safeguards and regulation credibility, contributing to the feeling of safety or danger across platforms (Kim et al., 2010).

From Figure 1, we see that all these pathways together form trust vulnerability with seniors, while we understand this as a relational exposure because it is the result of the interplay of individual skills, mental conditions, and institutional environments. The interpretation framework at the bottom of the figure, an empowerment–exposure balance, illustrates the double meaning of digital inclusion. The potential for digital literacy to increase participation and agency on the one hand, and risks and frauds through new vectors on the other hand, forms the conceptual underpinning of the hypotheses and the empirical tests below.

## 3. Methods

### 3.1. Data and Sample

This study adopts a quantitative, cross-sectional observational research design based on secondary analysis of nationally representative survey data. Specifically, this study uses data from the 2022 wave of the China Family Panel Studies (CFPS), a large-scale, nationally representative household survey conducted by the Institute of Social Science Survey at Peking University in China. The CFPS covers approximately 16,000 households and 40,000 individuals across 25 provinces and employs a multistage probability sampling strategy to capture information on demographics, education, health, income, family relations and digital activities. Owing to its design and scope, the CFPS provides a comprehensive basis for analysing social and economic change in contemporary China. The CFPS collects detailed information on individuals’ demographic characteristics, digital practices, social attitudes, and subjective well-being, making it particularly suitable for examining the relationship between digital literacy and trust-related outcomes among older adults.

The 2022 wave is particularly suited to the present analysis because it includes both behavioural indicators of digital participation and attitudinal measures of trust. Specifically, it contains items on short-video viewing, online learning, digital payment adoption and problem-solving via the internet, which can be mapped onto a multidimensional framework of digital literacy. It also includes a direct measure of trust in strangers (QN10024), which serves as a proxy for trust vulnerability. The analytic sample is restricted to respondents aged 60 and above. Observations with missing values or invalid skip codes are excluded, including cases indicating unfamiliarity with mobile payments. The final sample comprises 1583 older adults. As robustness checks, supplementary analyses are conducted for adult and youth subsamples. Descriptive statistics are summarised in Table 1. Analyses involving restricted variables (digital_trans, plant_security) were conducted in the restricted data room of the Institute of Social Science Survey, Peking University (CFPS).

### 3.2. Measurement of Key Variables

#### 3.2.1. Dependent Variable: Trust Vulnerability

Classical trust theory defines trust as a willingness to accept the potential vulnerability brought about by other people’s behaviour in a situation of uncertainty and incomplete information (Luhmann, 2018; Mayer et al., 1995). When this trust is extended to strangers in digital media interaction, it can be understood as a situational-dependent trust-related vulnerability due to limited verifiable clues (Ho & Hollister, 2013; Jones & Moncur, 2018; Lucassen & Schraagen, 2013; Thomson et al., 2015).

This interpretation is also consistent with the measurement method in international investigations and research. For example, comparative survey programmes such as the International Social Survey Programme (ISSP) assess online trust by asking respondents about their level of trust in “people who only communicate through the Internet and have never met offline” (Hadler et al., 2025; International Social Survey Programme [ISSP], 2023). This kind of survey design regards trust in unfamiliar network interaction objects as a trust orientation indicator under limited verification conditions.

Based on the above theory and measurement logic, this paper takes the self-reported trust level of respondents to strangers in CFPS 2022, QN10024, which requires respondents to evaluate their trust in strangers on a scale of 0–10, where 0 means “completely untrustworthy” and 10 means “completely trustworthy”. Therefore, higher scores mean that individuals are more inclined to express trust in unfamiliar others. In digital media interaction situations characterised by uncertainty and limited verification clues, this indicator can be understood as an individual’s tendency to extend trust in the face of unfamiliar interactive objects, thus acting as a proxy measure of trust-related vulnerabilities.

#### 3.2.2. Independent Variable: Digital Literacy

Digital literacy is defined as a multidimensional construct encompassing a broad set of cognitive and practical competences. In line with the DigComp 3.0 framework, digital competence comprises five interrelated domains: information and data literacy, communication and collaboration, digital content creation, safety, and problem-solving (Cosgrove & Cachia, 2025; Mattar et al., 2022; Nguyen et al., 2024; Rodríguez-Miranda et al., 2025). This mapping provides a more comprehensive and context-sensitive operationalisation than earlier CFPS-based studies that relied on narrower proxies such as internet-use frequency, mobile payment engagement, or smartphone ownership (Liu et al., 2021).

Since CFPS does not include performance-based skill tests, the measurements used in this article should be understood as behavioural agent indicators, not direct tests of technical skills. They reflect the digital abilities that the respondents have realised and can actually use in daily situations. On this basis, this paper constructs five-dimensional indicators and an overall digital literacy comprehensive index. The higher the value, the more extensive or more frequent the number of the interviewee’s participation in the corresponding dimension. For these five dimensions, please refer to Appendix A Table A1 for details on the corresponding CFPS question codes and their coding methods. In the follow-up analysis, this paper uses both comprehensive indexes and sub-dimensional indicators to portray the functional heterogeneity in different digital practices.

#### 3.2.3. Mediators

Subjective well-being is conceptualised as individuals’ overall cognitive and emotional evaluation of life, encompassing satisfaction, happiness, optimism and sense of purpose (Diener et al., 1985; Q. Zhang & Awaworyi Churchill, 2020). Drawing on CFPS 2022, a composite measure is constructed using five items: QM2016 (self-reported happiness, 0–10), QN12012 (life satisfaction, 1–5), QN12016 (confidence in the future, 1–5), QM3N (perceived life meaning, 0–10) and CES-D-based emotional support items (QN406–QN420), with reverse coding applied where appropriate. All variables are standardised and aggregated using principal component analysis (PCA), where higher values denote greater subjective well-being. This multidimensional approach aligns with recent CFPSs (Q. Zhang & Awaworyi Churchill, 2020) and provides a more nuanced depiction of psychological well-being than single-item metrics.

Platform security perception reflects users’ confidence in the financial safety and data protection of digital payment systems. CFPS items U12071 (perceived safety of money stored in mobile payment accounts) and U12072 (concerns about information leakage) are used to construct this measure. U12072 is reverse-coded, and both items are averaged so that higher values represent stronger perceived platform security. This operationalisation captures a salient dimension of digital trust in China, where mobile payments are deeply embedded in daily transactions.

#### 3.2.4. Control Variables

Control variables include demographic and socioeconomic characteristics commonly linked to trust and digital participation (Palmisano & Sacchi, 2024). Gender is coded as binary, age as continuous and hukou as agricultural versus non-agricultural. Education is converted into years of schooling. Household economic resources are measured by annual labour income, intergenerational transfers and relative income rank (1–5), with logged income used in regression models. Pension coverage, representing institutional security, is measured by receipt of retirement benefits or pension insurance. These controls adjust for heterogeneity that may jointly influence digital literacy, well-being and trust-related risk.

A comprehensive summary of variable definitions and measurement procedures, including item codes and data sources from CFPS 2022, is presented in Appendix A Table A1.

### 3.3. Empirical Models

We estimate the baseline association and mediation specifications as follows:

(1) $$Y_{i}=\alpha_{0}+\beta_{1}DL_{i}+X_{i}^{\prime }\gamma_{0}+\varepsilon_{i}$$

where $Y_{i}$ denotes individual *i*’s trust vulnerability, measured by generalised trust in strangers (higher scores indicate greater trust and hence stronger vulnerability). *DL_i_* is digital literacy, $X_{i}$ is a vector of demographic and socioeconomic controls, and $\varepsilon_{i}$ is an error term.

(2) $$M_{ki}=\alpha_{1k}+\beta_{2k}DL_{i}+X_{i}^{\prime }\gamma_{1k}+\varepsilon_{1ki}$$

(3) $$Y_{i}=\alpha_{2k}+\beta_{3k}DL_{i}+\delta_{k}M_{1k}+X_{i}^{\prime }\gamma_{2k}+\mu_{ki}$$

where $M_{ki}$ denotes the mediator, with k=1 referring to subjective well-being and k=2 referring to perceived platform security. Indirect effects are assessed using bias-corrected and accelerated (BCa) bootstrapping with 1000 replications implemented in Stata 18.0.

**H1** **(Direct corralation).** *Digital literacy will be positively correlated with trust vulnerability, reflecting empowerment with both efficiency- and vulnerability-increasing characteristics. Studies suggest that higher digital competence leads to increased exposure to digital risks (Aleti et al., 2025), while older adults are more susceptible to misleading information in low-verifiability environments, which heightens trust vulnerability (Brashier & Schacter, 2020).*

**H1a–H1e.**  *Each competence dimension—information, communication, content creation, transaction and problem-solving—is expected to show a positive association with trust vulnerability. Increased competence in domains like transactions and problem-solving can boost confidence, but also reduce caution, which may increased risk exposure in these areas (Aly et al., 2025; Zimba et al., 2025).*

**H2** **(Psychological mediation).** *Digital competence might also act indirectly on trust vulnerability through subjective well-being, because higher competence is associated with greater satisfaction and a stronger sense of control. Digital competence improves well-being, which can increase trust (Ding & Zhang, 2025). Internet engagement has also been found to enhance psychosocial outcomes like loneliness, suggesting well-being as a mediator in the competence-trust relationship (Murayama & Takase, 2025).*

**H3** **(Institutional mediation).** *Digital literacy may also be associated with trust vulnerability via trusted platforms, because elderly people who consider digital platforms safe are more likely to place trust in digital and interpersonal interactions. Perceived security has been shown to influence platform reliance (Clohessy et al., 2024), while trust in platform safety is also considered essential for digital adoption and continued use (Martinez et al., 2025; Niu et al., 2022), suggesting platform security as a mediator in the competence-trust relationship.*

**Figure 2:**
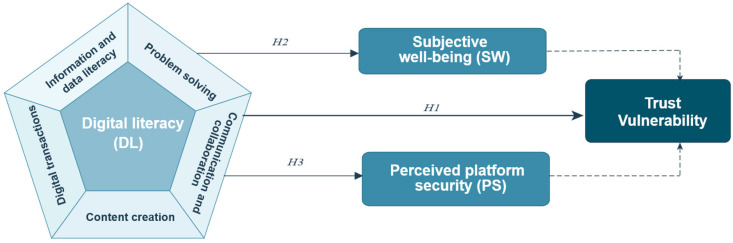
Conceptual framework linking digital literacy, mediators and trust vulnerability. Notes: The dashed panel lists the five dimensions of digital literacy used in the analysis: information (digital_infor), communication (digital_comm), content creation (digital_creat), transactions (digital_trans) and problem-solving (digital_prosolve). Solid arrows denote hypothesised relationships whereby digital literacy affects subjective well-being (SW) and perceived platform security (PS), which in turn influence trust vulnerability (TV). Control variables are omitted for clarity.

This integrated model (see Figure 2) provides the empirical foundation for the analyses that follow.

## 4. Results

The empirical analyses are structured around these research questions: Table 2 addresses RQ1, Table 3 examines mediation pathways (RQ2), and Table 4 presents heterogeneity analyses (RQ3).

### 4.1. Descriptive Statistics and Baseline Patterns

Table 1 summarises the descriptive characteristics of the variables used in the analysis. Trust vulnerability is measured by the survey item “trust in strangers” (QN10024) on a ten-point scale, where higher scores reflect a stronger inclination to trust and, consequently, greater potential exposure to uncertainty. The mean of 2.41 (SD = 2.30) indicates that older adults are concentrated toward the lower end of the scale, reflecting a generally cautious and risk-averse orientation towards unfamiliar others.

Life satisfaction (M = 4.16, SD = 0.82) and subjective well-being (M = 7.68, SD = 1.99) scored relatively high, reflecting that the respondents generally appraise their personal situations in a consistently positive manner. In contrast, perceived platform safety (M = 3.10, SD = 0.83) has a score close to the middle of the scale, and hence moderate trust in the safety of digital platforms and institutions’ safety measures. In this setting, low personal trust and medium institutional trust represent a cautious but active attitude towards social networking.

Significant differences appear in the dimensions of digital literacy. Communicative literacy receives the highest mean (M = 4.25, SD = 1.07), as messaging and social network apps are common for maintaining family and social connections. Transaction literacy (M = 1.37, SD = 1.26) and content creation literacy (M = 1.46, SD = 1.97) take mid-rankings, representing differential activity in mobile payment and online sharing activities, respectively. Problem-solving literacy (M = 0.35, SD = 0.54) reports the lowest average, reflecting ongoing challenges in confronting complex or novel digital activities. These patterns as a whole reveal that older adults’ digital behaviour remains largely communicative in character, with more functional competences maturing at a more leisurely rate.

For completeness, the pairwise correlations among all variables prior to regression analyses are reported in Appendix A Table A2.

### 4.2. Direct Associations of Digital Literacy

**Figure 3:**
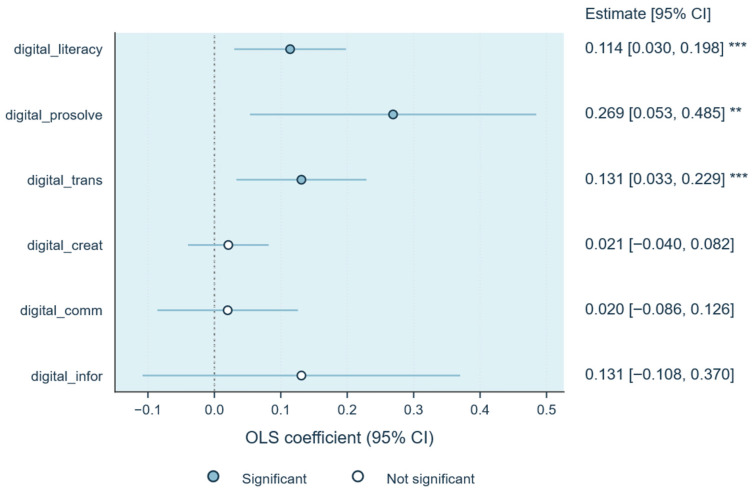
Direct associations of digital literacy on trust vulnerability (OLS estimates). Notes: Coefficients are plotted with 95 percent confidence intervals. Filled circles indicate statistically significant estimates, and open circles indicate non-significant associations. Models control for gender, age, education, income, hukou and pension status. *** *p* < 0.01, ** *p* < 0.05, * *p* < 0.1.

Baseline models for the elderly cohort produce a pattern of contrasts across the dimensions of digital literacy (Table 2; Figure 3). To illustrate the robustness of the estimated associations, we report baseline models without controls (Model 1) alongside fully adjusted models that include demographic and socioeconomic covariates (Model 2). On some of these dimensions, they are silent, barely skimming the surface of trust. When control variables are included, information literacy (β = 0.131, SE = 0.122) and communication literacy (β = 0.020, SE = 0.054) are both non-significant, with their confidence intervals wide and centred around zero. This pattern is confirmed by a visual trace in Figure 3: the lines gather on the baseline where access to information and daily online contact rarely extend beyond the circle of known faces. In this regard, online connection does not automatically broaden the horizon of social trust; it mainly reinforces these local connections.

Where informational skills remain still, the functional competences move sharply. Content creation literacy shows a significant correlation in the uncontrolled model (β = 0.089, SE = 0.030, *p* < 0.01), but the correlation is no longer significant after adding control variables (β = 0.021, SE = 0.031). In contrast, the correlation of digital transactions remains stable. It remains positive and significant in every specification (β = 0.183, SE = 0.046 without controls; β = 0.131, SE = 0.050 with controls). Figure 3 clearly shows this difference. The estimated point of transaction ability and problem-solving ability is located on the right side of 0, with a narrow confidence interval and relatively stable, while the content creation ability declines and approaches the neutral position.

Problem-solving skills emerge as having the highest and most persistent association across all dimensions (β = 0.379, SE = 0.104, *p* < 0.01; β = 0.269, SE = 0.110, *p* < 0.05 after controls). Elderly people who can independently complete complex digital tasks are more inclined to take the initiative and trust when dealing with uncertainty. This behaviour in turn strengthens their sense of self-efficacy and constitutes a positive feedback mechanism between digital empowerment and psychological confidence.

The pattern remains consistent when the focus is directed towards the composite index of digital literacy on trust vulnerability. The pattern holds when attention shifts to the composite index of digital literacy. The coefficient (β = 0.166, SE = 0.039, *p* < 0.01) points to a broader connection between digital competence and the willingness to trust amid uncertainty. In the visual field of Figure 3, the practical strands of literacy, transactional and problem-solving skills, together with the composite measure, lean distinctly to the right of the zero line, quietly separating themselves from the informational and communicative forms that hover around neutrality. The evidence moves in one direction: trust expands not through exposure to information but through the lived fluency of digital practice, the ordinary actions of navigating, paying and resolving that make risk feel manageable.

We conducted several diagnostic checks to assess the adequacy of the OLS specification. Residual–fitted value plots show no clear systematic patterns, suggesting that the linear specification is appropriate. Although residuals deviate moderately from normality, the relatively large sample size ensures the consistency of the estimates, and all models are estimated using Huber–White robust standard errors to obtain reliable inference. Diagnostics based on leverage statistics and Cook’s distance indicate that the results are not driven by influential observations, and variance inflation factors (maximum VIF = 1.36; mean VIF = 1.16) suggest that multicollinearity is minimal.

As a robustness check, we re-estimate the baseline models using clustered standard errors to account for the multistage sampling structure of the CFPS data. Specifically, we implemented clustering at the village/community level, clustering at the county/district level, and clustering at the village/community level with county/district fixed effects. The results remain highly consistent with the baseline estimates in terms of coefficient signs, magnitudes, and statistical significance, suggesting that the main findings are robust to potential clustering effects in the sampling design.

### 4.3. Mediation Analysis

Table 3 displays our mediation results of subjective well-being and perceived platform security for the mediating effects of digital literacy on trust vulnerability. Increased digital literacy is connected with higher levels of both mediators. People who score higher on their ability to handle digital media and devices also feel more satisfaction with their lives and are more confident that digital infrastructures are reliable. Estimated coefficients are β = 0.054 (SE = 0.028, *p* < 0.1) and β = 0.129 (SE = 0.016, *p* < 0.01). These relationships suggest that digital ability has components that do not simply reflect operational dexterity but show how people make sense of satisfaction and institutional trustworthiness in their everyday encounters with technologies.

The results from the mediation tests show a picture of high stability. Regardless of whether subjective well-being or perceived platform security is inserted into the model, the direct connection between digital literacy and trust vulnerability remains almost unaffected. In the first specification, where subjective well-being is controlled, the coefficient is β = 0.115 (SE = 0.043, *p* < 0.01). When the platform security variable is incorporated into the model, the correlation coefficient has hardly changed (β = 0.113, SE = 0.047, *p* < 0.01). This consistency of cross-model results shows that the relationship between digital skills and trust vulnerability is only slightly associated with psychological or institutional factors.

The mediating effect is relatively weak. The indirect effect of the transmission through subjective happiness is 0.022, and the indirect effect of the transmission of the sense of security through the platform is 0.005. The confidence intervals of both cross the zero point, indicating that their indirect effect is not statistically significant. From this, it can be inferred that the mechanism of digital literacy on the trust vulnerability of the elderly is mainly based on the direct behaviour path, and the intermediary effect at the emotional and institutional levels is relatively limited. The enhancement of digital proficiency has transformed the manner in which the elderly navigate the digital landscape, while also redefining their trust and risk assessment frameworks in ambiguous circumstances.

### 4.4. Heterogeneity Analysis

**Figure 4:**
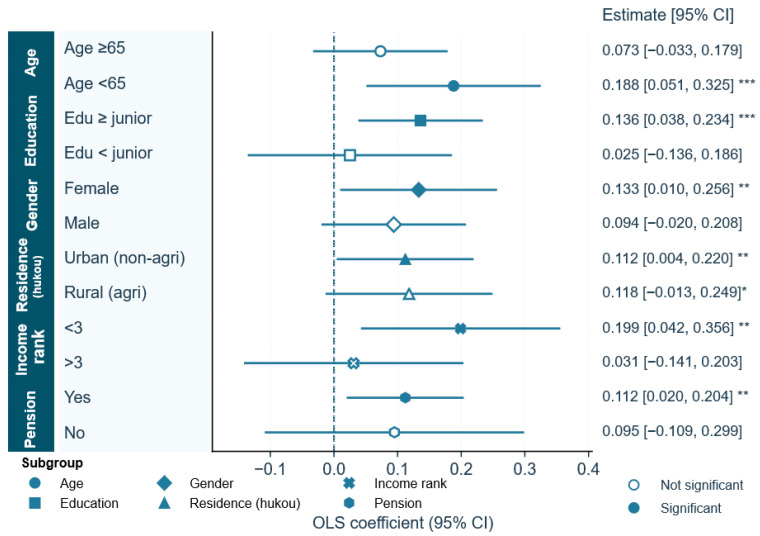
Heterogeneity of the association between digital literacy and trust vulnerability (elderly sample). Notes: Dot-and-whisker plots display OLS coefficients from subgroup regressions defined by age, education, gender, residence, income rank and pension status. Whiskers represent 95 percent confidence intervals. Solid markers indicate statistically significant estimates, and hollow markers denote non-significance. Each regression includes the standard control variables. *** *p* < 0.01, ** *p* < 0.05, * *p* < 0.1.

Table 4 presents the subgroup regressions and demonstrates that the association between digital literacy and trust vulnerability displays structured rather than random heterogeneity across demographic, socioeconomic and institutional contexts. Because numerical tables alone make it difficult to grasp the ordering, precision and overlap of these relationships, Figure 4 visualises the same results as a forest plot, aligning point estimates and their 95 percent confidence intervals on a common scale. The figure and the table complement each other, jointly illustrating a layered pattern of digital engagement in later life, where digital competence operates as both a capability and a site of exposure.

The age dimension provides the most straightforward benchmark. Descriptive statistics imply that digital literacy drops with age and that variation across the elderly is large. In the regression models, the younger-elderly (<65) display a positive and significant estimate (β = 0.188, SE = 0.070, *p* < 0.01), while the ≥65 category displays a weaker but non-significant estimate (β = 0.073, SE = 0.054, n.s.). This comparison is also visually prominent in Figure 4: the confidence interval for <65 is to the right of zero and narrow, but the one for ≥65 overlaps with zero. The point is that if improvements in digital competence contribute to an increasing trust orientation the benefit is particularly significant in the younger half of the older age group (where engagement is more spontaneous and consequently uncertainty is higher).

Differences in education emerge as one of the clearest dividing lines. Participants with at least junior schooling display a distinct positive link between digital competence and trust vulnerability (β = 0.136, SE = 0.050, *p* < 0.01). Those with lower educational attainment, however, show no comparable pattern (β = 0.025, SE = 0.082). In Figure 4, the band for the higher-educated group sits neatly to the right of zero, its narrow span signalling precision, while the interval for the less-educated group drifts across the baseline. This visual symmetry and contrast suggest that the transformation of digital skills to trust behaviour needs to be based on certain cognitive and operational abilities.

Gender disparities exhibit analogous patterns; however, they also possess distinct social attributes. The relationship is significant and beneficial for women (β = 0.133, SE = 0.063, *p* < 0.05); however, for men, the relationship is rather minor and statistically insignificant (β = 0.094, SE = 0.058). The forest map distinctly illustrates this disparity; the confidence interval for women is unequivocally positioned to the right of the zero point, whereas the confidence interval for men intersects the zero point. One possible interpretation is that older women may be more engaged in relational and communication-oriented online activities, which often rely more heavily on trust judgements. Prior research has documented gender differences in online communication styles and social media use, with women tending to place greater emphasis on relational interaction (Lin & Wang, 2020). Under digitally mediated conditions characterised by limited verification, this engagement may coincide with greater exposure to trust-related risks.

The socioeconomic environment formed by the difference between urban and rural areas and income further reveals the intertwining characteristics of digital opportunities and risk exposure. Whether it is urban (non-agricultural) or rural (agricultural) residents, their regression coefficients are positive (urban: β = 0.112, SE = 0.055, *p* < 0.05; rural: β = 0.118, SE = 0.067, *p* < 0.10). Figure 4 shows that the confidence interval between the two overlaps significantly, indicating that the gap between urban and rural areas is not significant. However, the difference in the income dimension is more obvious. The coefficient of the group in the lowest income tripart is large and significant (β = 0.199, SE = 0.080, *p* < 0.05), while the coefficient of the high-income group is smaller and not significant (β = 0.031, SE = 0.088). As can be seen in the figure, the confidence interval of low-income groups is firmly located on the right side of the zero point, while the confidence interval of high-income groups crosses the zero point. This outcome indicates that in contexts of constrained economic resources, the relationship of digital capabilities and trust vulnerability is more pronounced, and the everyday digital engagement of the elderly entails both inclusive opportunities and increased risk exposure.

Under the backdrop of varying social security and institutional trust, the relationship of digital literacy and trust vulnerability reveals fresh distinctions. The relationship is significant for pensioned respondents (β = 0.112, SE = 0.047, *p* < 0.05) and non-significant for those without pensions (β = 0.095, SE = 0.104). Figure 4 intuitively reflects this difference, with the confidence interval of the pension-receiving group lying entirely on the positive side, whereas that of the non-pension-receiving group crosses zero. The results suggest that enhancing institutional guarantees can improve the trust and participation of individuals in the digital environment. At the same time, their absence may be associated with increased uncertainty and vulnerability. This difference in trust, caused by institutional factors, reveals the complex interaction between digital ability and vulnerability in the digital lives of the elderly.

## 5. Discussion

### 5.1. Reinterpreting the Competence–Risk Paradox

Promoting digital skills is typically comprehended as a way to promote inclusion and empowerment. The data described here, however, are more complex. In fact, we observe that higher “numeracy” scores for older people are not associated with increased safety but with higher levels of trust in strangers, which we interpret here as greater situational trust vulnerability. Gaining technical expertise may be associated with greater self-confidence, but not necessarily with stronger risk awareness. Its spread is commonly accompanied by opaque or unbalanced online interaction, which will obfuscate people’s judgement more blurred in these situations. Enablement and vulnerability are both proximate products of the same phenomenon, the competency–threat imbalance that behavioural science refers to as the competence–risk effect. With the improvement of skills, people may exhibit more confidence and diminished apprehension online, but such confidence may also be associated with greater susceptibility to risk.

Previous studies have tended to portray digital literacy as a protective resource that reduces exclusion and encourages engagement (Rossi et al., 2024). The analysis of our study presents a more detailed perspective, showing that the association between digital capabilities and different dimensions is different. There is a weak positive correlation between digital literacy and trust susceptibility for problem-solving and online transactions, while the relationship of literacy and information processing and communication is weak or even not significant. Literacies for evaluating a situation or for transaction have a specific behavioural impact, and entail greater susceptibility to errors in trust (Rossi et al., 2024). While technical ability indicates skill in executing tasks, it may also be associated with greater willingness to trust beyond what careful judgement would warrant, possibly coinciding with lower risk perception and reduced vigilance. The ability of information processing and communication seems to exert a more restricted influence due to its profound integration into conventional communication practices. This further elucidates that digital vulnerability does not merely originate from “getting digital access”, but more from the way digital skills are practised and internalised in daily life.

Social structure reinforces this pattern. The association between competence and risk is more pronounced among older individuals, women, those with low income, and individuals with less education, reflecting a persistent inequality in digital participation (Helsper, 2012; Ragnedda et al., 2022). While functional literacy is associated with greater capacity to access information and engage in interaction, it is also associated with greater exposure to risk in a trust-based digital environment. Institutional-level resources are insufficient to mitigate this damage. Even with pension coverage and stable income, higher digital proficiency still correlates with greater risk (Aleti et al., 2025). These findings suggest that digital inclusion and digital risk evolve together within the same unequal social and institutional contexts.

In later life, functional independence does not eliminate vulnerability, but integrates into it. These abilities and skills should not be read as revealing a fixed trait of “vulnerability”; rather, they help us interpret how trust in strangers may reflect a situational form of vulnerability in the dynamics of everyday trust encounters. This situational vulnerability, as interpreted from trust in strangers, is not the antithesis of “trust” nor a singular or exhaustive characteristic of “trust” itself, but rather an interpretive dimension of trust under conditions of uncertainty in modern digital life.

### 5.2. Functional Versus Psychological Pathways

These characteristics suggest that digital capabilities may be associated with vulnerability through behavioural processes. Higher levels of digital transactions and problem-solving abilities are, to some degree, associated with higher levels of trust vulnerabilities. Although digital proficiency can improve efficiency and confidence, it may also convey the semblance of control beyond the genuine capacity for risk assessment. According to the theory of “ability orientation”, this result is reliant not on the individual’s ability level, but on the manner in which these talents are practiced and manifested in digital experiences.

From the perspective of Bandura’s theory of self-efficacy, the strengthening of functional ability not only enhances individuals’ belief in the correctness of their own behaviour, but also shapes a continuous illusion of control. The empirical findings of this study show that while functional ability might yield positive benefits and significance, it may coexist with forms of implicit overconfidence, which may in turn be associated with lower vigilance regarding potential risks. In this sense, the DigComp 3.0 framework further points out that the proficiency of digital capabilities is not equivalent to the improvement of risk protection capabilities. On the contrary, this kind of proficiency is likely to manifest as a “practiced routine,” wherein individuals unknowingly develop habitual patterns of digital behaviour that inform their logic of judgement, trust, and alertness, thereby sustaining a nuanced tension between participation and defence.

This pattern is also consistent with the limited mediating role of subjective well-being and perceived platform security. The situational trust vulnerability observed in this study appears to be linked less to a lack of comfort or institutional security than to their active engagement with digital platforms. Digital literacy has become profoundly embedded in individuals’ daily routines. While it may support more active participation, it may also coincide with new forms of risk under conditions of transparency and vulnerability. This phenomenon echoes the theory of “risk society” proposed by Beck, which often comes from systems aimed at controlling risks. In the digital era, abilities that foster a sense of belonging and engagement may concurrently heighten unseen hazards, as individuals progressively diminish their acute awareness of uncertainty while acquiring convenience and trust (Sundberg, 2024). The elderly group is the concentrated embodiment of this contradiction. Their digital participation may reduce exclusion to a certain extent, but may also be associated with greater dependence on digital infrastructure, which often becomes visible only through its consequences. From this perspective, inclusion and vulnerability coexist, participation is not an endpoint but an ongoing process of equilibrium and dialogue.

### 5.3. Policy Implications

The heterogeneity results suggest that structural position shapes how digital capabilities relate to risk exposure. The association between digital literacy and trust vulnerability is more pronounced among women and lower-income groups, possibly reflecting unequal digital opportunities. For these groups, improved capabilities may facilitate participation while increasing exposure to risk in contexts with limited protection. Pension coverage, while providing institutional stability, does not reduce trust vulnerability, suggesting that institutional security may increase confidence without enhancing vigilance.

These findings highlight a policy tension in digital inclusion. While improving digital capabilities promotes participation, it may also increase confidence in unfamiliar online interactions. Thus, empowerment and protection should be treated as complementary objectives. Digital education programmes may benefit from integrating risk awareness and verification practices alongside functional skills. Community-based training could include practical exercises, such as identifying suspicious messages, verifying online identities, and reflecting on decisions under uncertainty, to strengthen users’ risk management without discouraging participation (Rau & Premo, 2025; Lu et al., 2024; Schirmer et al., 2022).

The results also show that digital transaction and problem-solving capabilities are most consistently associated with higher trust in strangers, suggesting that transactional contexts involve greater exposure to low-verification interactions. Policy interventions may therefore prioritise these contexts by embedding verification prompts or decision checkpoints in payment, transfer, or service processes. Evidence from mobile money systems indicates that fraud often exploits routine behaviour, highlighting the importance of safeguards that interrupt automatic responses and encourage verification (Zimba et al., 2025). Interface-based warnings and decision-support tools may further help users identify risks while maintaining engagement (Hu et al., 2023).

Finally, the observed heterogeneity suggests that uniform training programmes may be insufficient. Subgroup-sensitive interventions may be more effective. For example, programmes targeting lower-income groups could emphasise digital financial practices and everyday risk recognition, particularly in platform-based environments (Choung et al., 2023). Scenario-based learning may improve judgement and reduce susceptibility to scams (Sur et al., 2023), while post-incident support, such as reporting channels and recovery guidance, can mitigate the consequences of victimisation.

### 5.4. Limitations and Directions for Future Research

There are still certain limits, even though our work broadens our awareness of the role that digital literacy plays in trust susceptibility and offers a fresh viewpoint for pertinent research. Given that the analysis is predicated on questionnaire survey data, it is indicative of the capacity of individuals to describe their own behaviour, rather than their actual behaviour. In addition, the indicators used to measure digital literacy are based on behavioural proxy measures derived from reported digital activities rather than direct tests of technical skills. The indicators utilised in this study can only show the expressiveness or ability dimension of digital literacy; they cannot cover the traits of the perceptual or relational level, despite the fact that CFPS data is nationally representative. Future research may consider combining questionnaires with behavioural tracking, experimental research or qualitative observation to further reveal the true presentation of trust vulnerability in daily online situations.

Cross-sectional data can reveal the correlation, but it is hard to draw causal inferences. The relationship between digital skills and risk alertness may be a psychological process that progressively changes as experience builds, rather than a straightforward strengthening or weakening. Future research using longitudinal data, panel designs, or experimental approaches would be valuable for examining the causal mechanism.

Digital inclusion is not merely an enhancement of capabilities, but a dynamic process of ongoing equilibrium between empowerment and exposure. This complexity underscores the necessity of examining security and trust processes inside the digital society from a more systematic viewpoint. Future study may investigate how platform design, governance systems, and social support might enhance or alleviate trust vulnerability in various contexts, while incorporating this topic within a wider discourse on ageing, digital literacy, and risk. In this sense, our study serves as a foundational exploration of digital inclusion as a social process characterised by ongoing creation and interactive evolution within the broader framework of “risk society”.

## 6. Conclusions

Through the analysis of the relationship between digital literacy and the trust vulnerability of the elderly, this article reveals the risk paradox contained in the process of digital empowerment. Improvements in digital capabilities, particularly in functional areas such as transactions and problem-solving, appear to support older adults’ engagement and participation in the digital society. However, greater proficiency does not necessarily translate into a stronger sense of security. With the improvement of operational proficiency, some elderly people are more likely to relax their vigilance and trust others excessively, thus falling into new risk situations. This finding shows that digital empowerment is not a one-way “gain process”, but at the same time harbours potential trust vulnerabilities, and its positive factors and risk factors coexist in the same mechanism.

This study further expands the theoretical connotation of digital literacy and reveals that the essence of digital inclusion is a dynamic balance process of continuous adjustment between autonomy and protection. Being familiar with and fluently using digital technology can reduce uncertainty and improve control, but it may also invisibly weaken individual risk awareness and critical judgement, making “empowerment” and “exposure” two sides of the same digital experience. This two-way mechanism shows that digital inclusion should not be regarded as a linear evolution from “access” to “empowerment”, but should be understood as a social practice process in which the elderly constantly seek a balance between trust and risk. This understanding is in line with the sociological theory of postmodern society on the interaction between trust and risk (Beck, 1992; Helsper, 2012; Sundberg, 2024), and provides theoretical support for the logic of re-examining the role of digital literacy in an ageing society.

From a practical point of view, the sustainable development of digital inclusion depends not only on the deepening of capacity building, but also on the embedding of security mechanisms and multi-party coordination. Education should focus on cultivating the critical thinking and reflective judgement of the elderly, so that they can maintain the necessary risk alertness in digital applications; system design should strengthen the visibility and verifiability of security mechanisms, and rebuild trust through transparency and understandability; and the coordination between individuals, platforms and public institutions should be committed to transforming capabilities into rational trust and common responsibility. In an ageing society, only by achieving the dual goals of empowerment and protection in the balance of “autonomy” and “care” can we truly promote the safe, equal and dignified integration of the elderly into the digital society.

## Figures and Tables

**Table 1:** Descriptive statistics of the elderly sample.

Variables	N	Mean	SD	Median	Min	Max
trust_vulnerability	1583	2.414	2.302	2	0	10
digital_infor	1583	0.75	0.482	1	0	2
digital_comm	1583	4.247	1.065	5	1	5
digital_creat	1583	1.459	1.967	0	0	6
digital_trans	1583	1.374	1.256	0	0	5
digital_prosolve	1583	0.345	0.539	0	0	2
digital_literacy	1583	−1.174	1.43	−1.343	−4.078	3.512
wellbeing	1583	7.678	1.993	8	0	10
satisfaction	1578	4.162	0.821	4	1	5
futureconf_e	1578	4.153	0.896	4	1	5
lifemeaning	1580	7.622	2.002	8	0	10
cespca	1568	0.226	1.795	0.608	−8.258	2.304
sw	1564	0.237	1.616	0.391	−6.526	2.582
plantform_security	1342	3.098	0.829	3	1	5
male	1583	0.557	0.497	1	0	1
age	1583	66.637	5.321	66	60	88
hukourural	1582	0.496	0.5	0	0	1
edu	1583	8.373	4.422	9	0	19
income	1583	3220.666	5515.653	2316.667	0	112,000
income_rank	1562	3.027	1.046	3	1	5
pension_insured	1580	0.812	0.391	1	0	1

Notes: Values represent means, standard deviations (SD), medians, minima, and maxima for all variables used in the analysis. Statistics are based on the elderly subsample aged 60 years and above from the China Family Panel Studies (CFPS) 2022 (N = 1583). Variable definitions and coding schemes are detailed in Appendix A Table A1.

**Table 2:** Baseline OLS estimates of digital literacy and trust vulnerability among older adults.

Variables	(1)	(2)	(3)	(4)	(5)	(6)	(7)	(8)	(9)	(10)	(11)	(12)
	Trust Vulnerability No Controls	Trust Vulnerability + Controls	Trust Vulnerability No Controls	Trust Vulnerability + Controls	Trust Vulnerability No Controls	Trust Vulnerability + Controls	Trust Vulnerability No Controls	Trust Vulnerability + Controls	Trust Vulnerability No Controls	Trust Vulnerability + Controls	Trust Vulnerability No Controls	Trust Vulnerability + Controls
digital_infor	0.035	0.131										
	(0.123)	(0.122)										
digital_comm			0.030	0.020								
			(0.053)	(0.054)								
digital_creat					0.089 ***	0.021						
					(0.030)	(0.031)						
digital_trans							0.183 ***	0.131 ***				
							(0.046)	(0.050)				
digital_prosolve									0.379 ***	0.269 **		
									(0.104)	(0.110)		
digital_literacy											0.166 ***	0.114 ***
											(0.039)	(0.043)
male		0.607 ***		0.605 ***		0.599 ***		0.579 ***		0.622 ***		0.602 ***
		(0.118)		(0.118)		(0.118)		(0.118)		(0.118)		(0.118)
age		0.029 **		0.028 **		0.027 **		0.034 ***		0.029 **		0.032 ***
		(0.012)		(0.011)		(0.011)		(0.012)		(0.011)		(0.012)
hukourural		−0.369 ***		−0.357 ***		−0.348 **		−0.301 **		−0.311 **		−0.308 **
		(0.137)		(0.137)		(0.138)		(0.138)		(0.138)		(0.137)
edu		0.029 *		0.029 *		0.028 *		0.020		0.023		0.020
		(0.015)		(0.015)		(0.015)		(0.015)		(0.015)		(0.015)
income		0.000		0.000		0.000		0.000		0.000		0.000
		(0.000)		(0.000)		(0.000)		(0.000)		(0.000)		(0.000)
income_rank		0.272 ***		0.270 ***		0.270 ***		0.274 ***		0.274 ***		0.269 ***
		(0.058)		(0.057)		(0.058)		(0.058)		(0.058)		(0.058)
pension_insured		0.194		0.200		0.191		0.191		0.178		0.180
		(0.151)		(0.151)		(0.151)		(0.151)		(0.150)		(0.151)
constant	2.388 ***	−1.022	2.285 ***	−0.939	2.285 ***	−0.817	2.162 ***	−1.356 *	2.283 ***	−1.019	2.608 ***	−0.946
	(0.110)	(0.814)	(0.225)	(0.852)	(0.071)	(0.795)	(0.086)	(0.810)	(0.069)	(0.801)	(0.073)	(0.795)
N	1583	1558	1583	1558	1583	1558	1583	1558	1583	1558	1583	1558
R_squared	0.000	0.062	0.000	0.061	0.006	0.061	0.010	0.065	0.008	0.064	0.011	0.065

Notes: Robust standard errors are reported in parentheses. All models control for gender, age, education, income, hukou, and pension status. *** *p* < 0.01, ** *p* < 0.05, * *p* < 0.1.

**Table 3:** Mediation of subjective well-being and perceived platform security.

Variables	(1) Trust Vulnerability	(2) Subjective Well-Being	(3) Trust Vulnerability	(4) Trust Vulnerability	(5) Platform Security	(6) Trust Vulnerability
Subjective well-being			0.022 (0.036)			
Digital literacy index	0.114 *** (0.043)	0.054 * (0.028)	0.115 *** (0.043)	0.114 *** (0.043)	0.129 *** (0.016)	0.113 ** (0.047)
Platform security						0.005 (0.083)
Constant	−0.946 (0.795)	−1.528 *** (0.545)	−0.991 (0.798)	−0.946 (0.795)	3.105 *** (0.321)	−1.252 (0.916)
Observations	1558	1544	1544	1558	1324	1324
R-squared	0.065	0.138	0.067	0.065	0.052	0.067
Controls	YES	YES	YES	YES	YES	YES

Notes: Robust standard errors are reported in parentheses. All models control for gender, age, education, income, hukou, and pension status. Columns (2) and (5) present the first-stage regressions of digital literacy on the mediators (subjective well-being and perceived platform security). Columns (3) and (6) include both mediators in the main regression predicting trust vulnerability. *** *p* < 0.01, ** *p* < 0.05, * *p* < 0.1.

**Table 4:** Heterogeneity of digital literacy and trust vulnerability (elderly sample).

Block	Subgroup	β (SE)
Age	Age ≥ 65	0.073 (0.054)
	Age < 65	0.188 *** (0.070)
Education	Edu ≥ junior	0.136 *** (0.050)
	Edu < junior	0.025 (0.082)
Gender	Female	0.133 ** (0.063)
	Male	0.094 (0.058)
Residence (hukou)	Urban (non-agri)	0.112 ** (0.055)
	Rural (agri)	0.118 * (0.067)
Income rank	<3	0.199 ** (0.080)
	>3	0.031 (0.088)
Pension	Yes	0.112 ** (0.047)
	No	0.095 (0.104)

Notes: OLS coefficients are reported for the composite digital literacy index, with robust standard errors in parentheses. Each regression includes the standard demographic and socioeconomic control variables. Full subgroup regression coefficients are presented in Appendix A Table A4. *** *p* < 0.01, ** *p* < 0.05, * *p* < 0.1.

## Data Availability

The public-use data employed in this study are available for download from the official China Family Panel Studies (CFPS) website (https://cfpsdata.pku.edu.cn/#/home) upon user registration. Access to the restricted-use CFPS data referenced in this paper requires a formal application to the CFPS project team. Restricted data may only be accessed and analysed within the designated CFPS data facility; the data are not publicly disclosed.

## Abbreviation

The following abbreviation is used in this manuscript:

CFPS	China Family Panel Studies

## Appendix A

**Table A1:** Variable Definitions and Measurement (CFPS 2022).

Variable Type	Variable Name (CFPS Code)	Definition	Measurement and Coding Description
Dependent variable	trust_strangers (QN10024)	Trust in strangers, measured on a 0–10 scale where higher scores indicate stronger trust and thus greater trust vulnerability.	QN10024 asks, “How much do you trust strangers?” (0 = cannot be trusted at all, 10 = can be fully trusted). Used as a proxy for trust vulnerability.
Independent variables (Digital literacy dimensions)	digital_infor (U93/U931; U94/U941)	Information and data literacy: frequency of short-video viewing and online learning.	U93, U931; U94, U941. Each daily activity = 1, otherwise = 0, summed to form a 0–2 index. Higher scores denote broader and more frequent information engagement.
	digital_comm (QN953)	Communication and collaboration: perceived importance of the Internet for maintaining contact with family and friends.	QN953 rated on a 1–5 scale (1 = not important, 5 = very important). Higher values indicate stronger reliance on digital communication.
	digital_creat (U111)	Digital content creation: frequency of posting or sharing on WeChat Moments.	U111 records frequency of posting on WeChat Moments. Higher values indicate greater frequency of content creation.
	digital_trans (U1201–U1205)	Digital transactions: proficiency in using mobile payment platforms such as WeChat Pay and Alipay.	U1201–U1205 measure use and weekly frequency of payments. The mean of frequency items (U1204–U1205) forms a 1–5 scale where higher values denote greater transactional fluency.
	digital_prosolve (U92/U921)	Problem-solving competence: ability to use the Internet to address practical needs.	U92 (online shopping) and U921 (daily online shopping). Each daily activity = 1, otherwise = 0; summed to 0–2 index.
	digital_literacy (composite index)	Overall digital literacy level, integrating the five dimensions above.	Standardised dimension scores combined using principal component analysis (PCA); the first component retained as the composite digital literacy index.
Mediators	SW (PCA composite)	Subjective well-being and perceived safety index.	Constructed using QM2016 (happiness, 0–10), QN12012 (life satisfaction, 1–5), QN12016 (confidence in the future, 1–5), QM3N (life meaning, 0–10), and CES-D items (QN406–QN420, reverse-coded where appropriate). All standardised and aggregated using PCA.
	plant_security (U12071/U12072)	Perceived platform security: confidence in financial safety and data protection of mobile payment systems.	Mean of U12071 (“fund safety”) and reverse-coded U12072 (“concern about information leakage”). Higher scores indicate stronger perceived platform security.
Control variables	male (QA002)	Gender.	1 = male, 0 = female.
	age	Age in years.	Continuous variable.
	hukou_rural (QA301)	Rural hukou registration.	1 = rural, 0 = non-rural.
	edu (KW01)	Years of schooling.	Converted from highest educational attainment.
	income_rank (QN8011)	Self-rated income position.	QN8011: 1 = very low … 5 = very high.
	pension_insured(QI200/QI2001)	Pension coverage.	1 = receives retirement benefits or pension insurance, 0 = none.

**Table A2:** Spearman correlation matrix of digital literacy, trust vulnerability, and control variables.

	trust_vulnerability	digital_infor	digital_comm	digital_creat	digi-tal_prosolve	digital_literacy	gender	age	ruralhukou	edu	incomerank	pension_insured
trust_vulnerability	1.000											
digital_infor	−0.003	1.000										
digital_comm	−0.035	0.077 ***	1.000									
digital_creat	0.089 ***	0.117 ***	0.073 ***	1.000								
digi-tal_prosolve	0.101 ***	0.173 ***	0.099 ***	0.267 ***	1.000							
digital_literacy	0.077 ***	0.561 ***	0.422 ***	0.663 ***	0.633 ***	1.000						
gender	0.148 ***	−0.027	−0.051**	0.076 ***	−0.016	0.007	1.000					
age	0.073 ***	−0.144 ***	−0.117 ***	0.024	−0.058 **	−0.103 ***	0.057 **	1.000				
ruralhukou	−0.121 ***	0.092 ***	0.000	−0.243 ***	−0.266 ***	−0.190 ***	0.014	−0.174 ***	1.000			
edu	0.123 ***	0.013	0.057 **	0.256 ***	0.298 ***	0.269 ***	0.172 ***	−0.134 ***	−0.405 ***	1.000		
incomerank	0.133 ***	−0.010	0.051 **	0.034	−0.018	0.034	0.061 **	0.037	0.017	0.001	1.000	
pension_insured	0.078 ***	−0.033	−0.028	0.123 ***	0.106 ***	0.085 ***	0.001	0.234 ***	−0.246 ***	0.137 ***	0.060 **	1.000

Notes: *** *p* < 0.01, ** *p* < 0.05, * *p* < 0.1; Due to data access restrictions, variables related to online security are classified as restricted-use data and can only be accessed within the CFPS secure data facility. Given the limited revision timeframe, the composite digital literacy index in this table is constructed using the first four observable dimensions only. This does not involve restricted data.

**Table A3:** Baseline OLS estimates of the association between digital literacy (equal-weighted mean index) and trust vulnerability among older adults.

Variables	Trust_strangers
digital_literacy_mean	0.076 *
	(0.040)
gender	0.266 ***
	(0.052)
age	0.013 ***
	(0.005)
ruralhukou	−0.155 ***
	(0.058)
edu	0.011 *
	(0.007)
incomerank	0.117 ***
	(0.025)
pension_insured	0.083
	(0.066)
Constant	−1.577 ***
	(0.349)
Observations	1559
R-squared	0.063

Notes: *** *p* < 0.01, ** *p* < 0.05, * *p* < 0.1. Due to data access restrictions, variables related to online security are classified as restricted-use data and can only be accessed within the CFPS secure data facility. Given the limited revision timeframe, the composite digital literacy index in this table is constructed using the first four observable dimensions only. This does not involve restricted data.

**Table A4:** Subgroup regressions: full coefficients (with controls).

Subgroup Dimension	Variable	Group 1 (β (SE))	Group 2 (β (SE))
Gender (Male/Female)	digital_infor	−0.009 (0.160)	0.327 * (0.186)
	digital_trans	0.142 ** (0.067)	0.112 (0.075)
	digital_probsovle	0.324 ** (0.153)	0.179 (0.158)
	digital_literacy	0.094 (0.058)	0.133 ** (0.063)
Age (≥65/<65)	digital_trans	0.065 (0.066)	0.235 *** (0.076)
	digital_probsovle	0.125 (0.146)	0.461 *** (0.167)
	digital_literacy	0.073 (0.054)	0.188 *** (0.070)
Hukou (Rural/Non-rural)	digital_trans	0.104 (0.074)	0.151 ** (0.068)
	digital_probsovle	0.325 * (0.182)	0.233 * (0.137)
	digital_literacy	0.118 * (0.067)	0.112 ** (0.055)
Education (≥Junior/<Junior)	digital_trans	0.122 ** (0.058)	0.132 (0.098)
	digital_probsovle	0.343 *** (0.124)	−0.178 (0.236)
	digital_literacy	0.136 *** (0.050)	0.025 (0.082)
Income rank (<3/>3)	digital_trans	0.250 *** (0.091)	−0.019 (0.097)
	digital_probsovle	0.428 ** (0.204)	−0.025 (0.247)
	digital_literacy	0.199 ** (0.080)	0.031 (0.088)
Pension coverage (Yes/No)	digital_trans	0.152 *** (0.055)	−0.001 (0.119)
	digital_probsovle	0.203 * (0.120)	0.632 ** (0.262)
	digital_literacy	0.112 ** (0.047)	0.095 (0.104)

Notes: *** *p* < 0.01, ** *p* < 0.05, * *p* < 0.1.
